# Nobiletin alleviates atherosclerosis by inhibiting lipid uptake via the PPARG/CD36 pathway

**DOI:** 10.1186/s12944-024-02049-5

**Published:** 2024-03-11

**Authors:** Heng Wang, Qinqin Tian, Ruijing Zhang, Qiujing Du, Jie Hu, Tingting Gao, Siqi Gao, Keyi Fan, Xing Cheng, Sheng Yan, Guoping Zheng, Honglin Dong

**Affiliations:** 1 Department of Vascular Surgery, The Second Hospital of Shanxi Medical University, Taiyuan, Shanxi China; 2 Department of Nephrology, The Second Hospital of Shanxi Medical University, Taiyuan, Shanxi China; 3 Jiangyin People’s Hospital, Wuxi, Jiangsu China; 4 Shanxi Bethune Hospital, Third Hospital of Shanxi Medical University, Shanxi Academy of Medical Sciences, Tongji Shanxi Hospital, Taiyuan, Shanxi China; 5 Centre for Transplant and Renal Research, Westmead Institute for Medical Research, The University of Sydney, Sydney, NSW Australia

**Keywords:** Atherosclerosis, Nobiletin, Network pharmacology, PPARG/CD36 pathway, Macrophagocyte, Inflammation

## Abstract

**Background:**

Atherosclerosis (AS) is a persistent inflammatory condition triggered and exacerbated by several factors including lipid accumulation, endothelial dysfunction and macrophages infiltration. Nobiletin (NOB) has been reported to alleviate atherosclerosis; however, the underlying mechanism remains incompletely understood.

**Methods:**

This study involved comprehensive bioinformatic analysis, including multidatabase target prediction; GO and KEGG enrichment analyses for function and pathway exploration; DeepSite and AutoDock for drug binding site prediction; and CIBERSORT for immune cell involvement. In addition, target intervention was verified via cell scratch assays, oil red O staining, ELISA, flow cytometry, qRT‒PCR and Western blotting. In addition, by establishing a mouse model of AS, it was demonstrated that NOB attenuated lipid accumulation and the extent of atherosclerotic lesions.

**Results:**

(1) Altogether, 141 potentially targetable genes were identified through which NOB could intervene in atherosclerosis. (2) Lipid and atherosclerosis, fluid shear stress and atherosclerosis may be the dominant pathways and potential mechanisms. (3) ALB, AKT1, CASP3 and 7 other genes were identified as the top 10 target genes. (4) Six genes, including PPARG, MMP9, SRC and 3 other genes, were related to the M0 fraction. (5) CD36 and PPARG were upregulated in atherosclerosis samples compared to the normal control. (6) By inhibiting lipid uptake in RAW264.7 cells, NOB prevents the formation of foam cell. (7) In RAW264.7 cells, the inhibitory effect of oxidized low-density lipoprotein on foam cells formation and lipid accumulation was closely associated with the PPARG signaling pathway. (8) In vivo validation showed that NOB significantly attenuated intra-arterial lipid accumulation and macrophage infiltration and reduced CD36 expression.

**Conclusions:**

Nobiletin alleviates atherosclerosis by inhibiting lipid uptake via the PPARG/CD36 pathway.

**Supplementary Information:**

The online version contains supplementary material available at 10.1186/s12944-024-02049-5.

## Introduction

Cardiovascular diseases are the most prevalent noncommunicable conditions globally and stand as the primary cause of death, making a significant contribution to disability worldwide [1, 2]. Atherosclerosis (AS) is the predominant pathological process marked by accumulated lipids and inflammation in the wall of large arteries, and a myocardial infarction or stroke could occur as a consequence [3, 4]. Current medications for atherosclerosis can only improve symptoms, cannot cure them completely, and may even lead to cardiovascular events when the medication is discontinued [5, 6]. Therefore, it is important to investigate the mechanism of atherosclerotic damage and find drugs that slow or reverse the progression of atherosclerotic vascular disease.

Recent studies have revealed that atherosclerosis is a pathological process involving multiple changes in the immune system, vascular wall-resident cells, genetic factors, hemodynamics, and lipid metabolism [7–10]. Although the etiology of atherosclerosis is not fully understood, the inflammatory polarization of macrophages induced by abnormal lipid accumulation is crucial in the progression of atherosclerosis [7, 11]. As the disease progresses, excessive levels of lipids in blood are deposited on the intima, and macrophages polarize into M1-type proinflammatory cells that phagocytose excess lipids into foam cells, promoting the formation of an inflammatory and necrotic core [12, 13]. In addition, macrophages have been associated with atherosclerotic plaque vulnerability [14]. Therefore, macrophages are important targets for atherosclerotic plaque detection and treatment [15, 16].

Nobiletin (NOB) is a polymethoxylated flavonoid existed in citrus peel [17]. A number of traditional Chinese medicines contain this bioactive molecule, including *Centipedae Herba, Citrus Reticulata, and Tripterygii Radix* [18]. Studies have shown that numerous benefits including anti-inflammation [19], anti-aging [20], antioxidant [21] and anti-tumor [22] can be derived from NOB. In addition, recent evidence suggests that NOB can improve dyslipidemia and ameliorate atherosclerosis, but the exact mechanism of action is not fully understood [23–25]. These findings imply that NOB could have the capacity for application in the clinical management of atherosclerosis.

Network pharmacology is a concept that many effective drugs modulate multiple targets, instead of specific one, which was presented by Hopkins in 2007 [26]. Network pharmacology combines systems medicine with information science as an advanced tool for drug discovery and development [27]. It is an approach that integrates computerized information to construct networks of “protein-compound/disease-gene,” revealing the synergistic mechanisms of action of conventional drugs [28, 29].

**Fig. 1:**
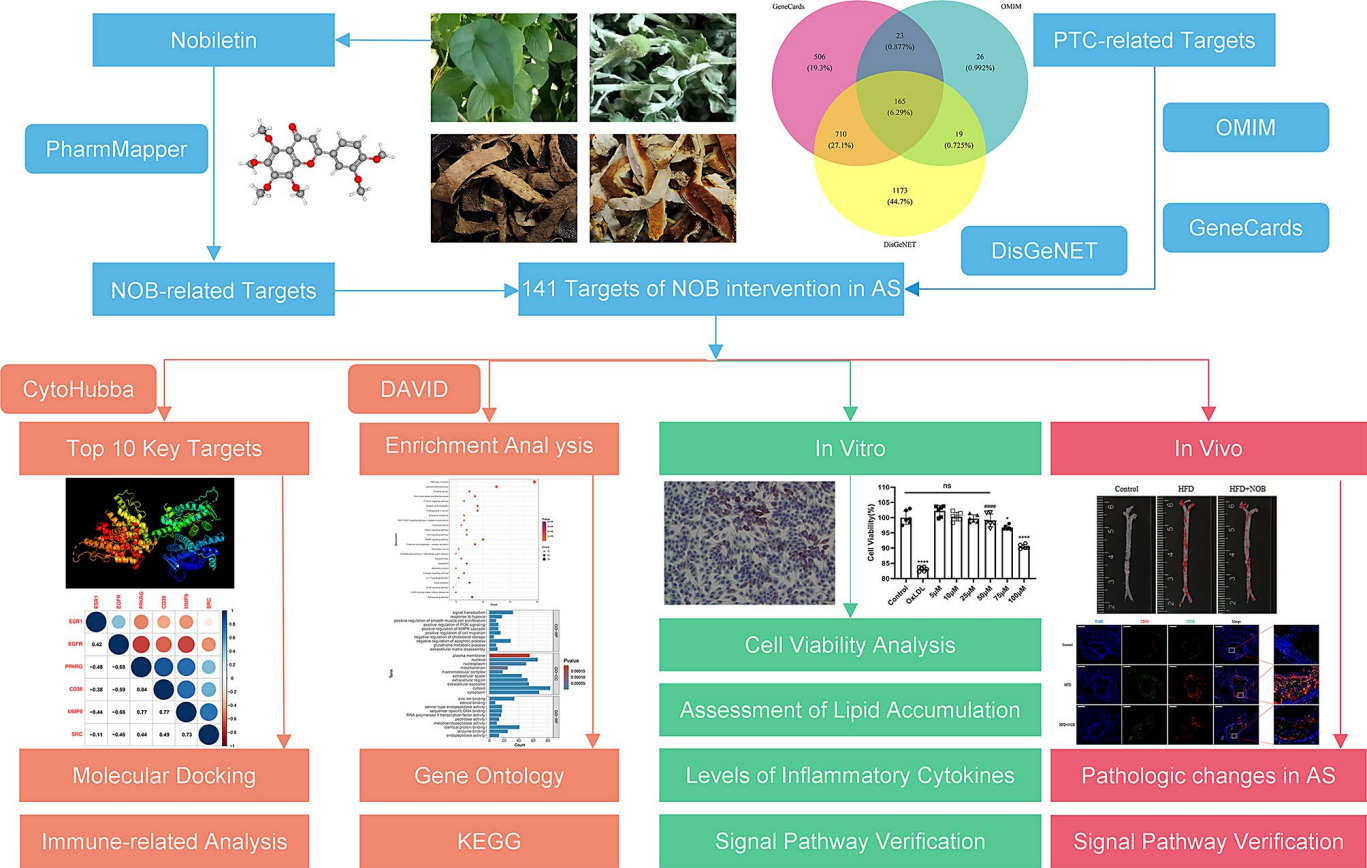
A flow chart illustrated the idea of the project. All photos of traditional Chinese medicines (*Centipedae Herba, Citrus Reticulata, Citri Reticulatae Pericarpium and Tripterygii Radix*) were taken recently, and the copyright is not disputed

This study is the first to discover the potential targets of NOB intervention in atherosclerosis through network pharmacological analysis. In vivo and ex vivo experiments were conducted to figure out whether NOB inhibits lipid uptake and the progression of atherosclerosis by modulating the PPARG/CD36 signaling pathway. Oxidized low-density lipoprotein (OxLDL)-treated mouse macrophages (RAW264.7) were used as an ex vivo model for atherosclerosis. This approach was used to corroborate the mechanism through which NOB modulates foam cell formation, the inflammatory response and apoptosis. Furthermore, NOB was found to reduce macrophage infiltration and lipid accumulation in atherosclerotic mice. This study revealed that NOB inhibits foam cells formation and attenuates the inflammatory reaction by regulating the peroxisome proliferator-activated receptor-γ (PPARG/PPAR-γ) signaling pathway. Figure 1 shows the flowchart of the experiment.

## Materials and methods

### Network pharmacology and molecular docking analysis

#### Targets screening for NOB and atherosclerosis

The PharmMapper Server [30] is a website help to discover potential targets for small molecule compounds, supported by a large database of pharmacodynamics. Drug-related targets were acquired from PharmMapper. Disease-related targets were downloaded from the following databases: GeneCards [31], OMIM [32] and DisGeNET [33]. Potential targets for NOB intervention in atherosclerosis were obtained by intersection analysis.

#### Enrichment analysis

With the functional annotation tool, researchers can use the DAVID bioinformatics resource to analyze the biological significance behind a list of genes [34]. Functions and pathways of possible targets of NOB intervention in atherosclerosis were analyzed using DAVID, and the R package ggplot2 was utilized to visualize the data. GO and KEGG analyses were performed to investigate the correlation of functions and pathways. The mechanism diagram in this paper was constructed in Figdraw (ID: IWTPTaca7a).

#### Identification of key targets based on protein-protein interaction (PPI) networks

Setting the minimum required interaction score as the medium confidence interval, the resulting PPI network was exported from STRING [35]. Based on the protein interactions, a drug-disease network diagram of NOB intervention in atherosclerosis was drawn in Cytoscape 3.9.0 [36]. The important and weak parts of the CytoHubba plugin in Cytoscape network were identified, indicating which targets may play significant roles. The algorithms used were as follows: degree, betweenness, closeness, maximum neighborhood component (MNC), edge percolated component (EPC) and maximal clique centrality (MCC).

#### Molecular docking

Ligand structure files could be found from the PubChem. Protein receptor structures [37–45] were downloaded from the RCSB Protein Data Bank (PDB). It is known that predicting druggable binding sites is crucial to structure-based drug design. DeepSite [46], a forecasting tool with advanced algorithms, was used for binding site prediction in this study. With the calculated Grid Box, AutoDock Vina 1.2.3 was used to molecular docking analysis [47]. Using PLIP [48] and PyMOL [49], this research explored the noncovalent interactions between biological macromolecules and NOB and visualized the results.

#### Correlation analysis of key gene and the macrophage fraction in patients’ samples of atherosclerosis

The expression profiles of 32 patients in GSE43292 [50] were obtained from the GEO database [51]; these included 32 carotid atheroma plaques and 32 macroscopically intact carotid tissues adjacent to the atheroma plaques. The percentage of immune cells in atherosclerotic plaques was calculated from the expression profile by the CIBERSORT algorithm. The macrophage fraction and key gene expression data were extracted separately to complete correlation analysis via the Spearman method. Furthermore, it was examined that the correlation between the key genes and macrophages.

### Ex vivo experiments

#### Cell culture

RAW264.7 cells (KeyGen Biotech, Nanjing, China) were cultured using DMEM medium containing 10% fetal bovine serum (FBS) and 1% penicillin-streptomycin. The temperature was 37 °C and the CO_2_ concentration was 5%. Cell proliferation was monitored by changing the medium every two days. RAW264.7 cells were intervened with OxLDL (Yiyuan Biotechnology, Guangzhou, China) to establish an ex vivo cellular model of atherosclerosis. The remaining reagents were purchased from Gibco.

#### Cell counting Kit-8 assay

NOB in a series of concentrations (0, 5, 10, 25, 50, 75 and 100 µM) were set to directly treat RAW264.7 cells to determine the optimal intervention dose of NOB (MedChemExpress, New Jersey State, USA). In order to determine the viability of cells in the presence of NOB, different concentrations of NOB were added to the culture, and RAW264.7 cells were incubated with OxLDL (50 µg/mL) [52] for 48 h. After that, 10 µL of Cell Counting Kit-8 (CCK-8) solution was introduced to the cells (Yeasen Biotechnology, Shanghai, China). Cell viability was assayed by measuring optical density (OD) at 450 nm after incubation for 2 h at 37 °C under light-avoidance conditions.

#### Cell scratch test

Inoculate a cell suspension to achieve a density of 1.0 × 10^5^ cells/mL into a 24-well plate at 0.5 mL per well. After waiting for the cells to attach to the wall, draw 3 scratches perpendicular to the bottom of the 24-well plate using narrow end of a 10 µL pipette. Phosphate buffered saline (PBS) was used to rinse the well plate 2–3 times, and suspended cells from the middle of the scratches can be washed away. The cells were arranged into the specified groupings: blank control, OxLDL (50 µg/mL), OxLDL + NOB (10 µM), OxLDL + NOB (50 µM) and NOB (50 µM). Images were taken after 48 h of intervention. The width of the scratch was measured using Image-Pro Plus software.

#### Oil red O staining

RAW264.7 cells were inoculated in 12-well plates to a density of 2 × 10^5^ cells/mL. The cells from different groups were fixed with oil Red O fixative (Solarbio, Beijing, China) for 25 min. After washing by PBS and 60% isopropanol, the nuclei received a 15-minute stain with a freshly mixed oil red O solution and then restained with cytohematoxylin staining solution for 1 min. Distilled water was added to cover the cells, which were subsequently observed under a microscope (Olympus, Tokyo, Japan). Anhydrous ethanol was added to extract the lipids that accumulated in the cells. Then, measure the absorbance at 50 nm using an enzyme marker to quantify the extent of foam cell formation.

#### Determination of TC and TG

After 200 µL of anhydrous ethanol was added to each group, the homogenate was crushed by ultrasonication under ice bath conditions and assayed directly by an enzyme marker without centrifugation. Total cholesterol (TC) and triglyceride (TG) levels in the cells were measured following the kits’ protocols (Jiancheng Institute, Nanjing, China).

#### ELISA

The cultivation of RAW264.7 cells was conducted as previously detailed by adding cell culture medium containing control medium, OxLDL (50 µg/mL), or OxLDL + NOB (10 or 50 µM). After 48 h of incubation, the supernatant above the cells was gathered. The levels of CCL2 and IL-6 were assessed utilizing ELISA kits following the manufacturer’s protocols (Ruixin Biotech, Quanzhou, China).

#### Flow cytometry assay

RAW264.7 cells were assayed using the Annexin V-FITC Apoptosis Detection Kit and then analyzed using flow cytometry (BD Biosciences, Franklin Lakes, NJ, USA).

#### Quantitative real-time PCR (qRT-PCR)

RAW264.7 cells were grouped and total RNA was extracted from the cells 48 h after the intervention on the cells (Mei5 Biotechnology, Beijing, China). Total RNA was reverse transcribed into cDNA using PrimeScript™ RT Master Mix (Takara Bio, Inc., Beijing, China). The cDNA was put on the machine (Thermo Fisher Scientific, Waltham, MA, USA) and assayed by a fluorescence quantification kit (Mei5 Biotechnology, Beijing, China). The expression of 10 genes (ALB, AKT1, CASP3, EGFR, SRC, MMP9, IGF1, HSP90AA1, PPARG, and ESR1) was normalized to the expression of ACTB. The 2^−∆∆Ct^ method was applied to conduct the data analysis, and the sequences for the primers were mentioned in Table 1.

#### Western blotting (WB) assay

Cells were treated with lysis buffer and denatured by boiling to obtain total protein. After concentration and separation on an SDS-PAGE gel, the proteins were transferred to a polyvinylidene difluoride (PVDF) membrane. Protein expression was then detected with the following primary antibodies: CD36 (Abcam, London, UK), PPARG (Proteintech, Wuhan, China), and GAPDH (Servicebio, Wuhan, China). Primary antibodies were incubated overnight at 4 °C, followed by 1.5 h of secondary antibody incubation. Finally, the cells were rinsed with ELC detection reagents for 30 s and analyzed on a ChemiDoc System (Bio-Rad, Shanghai, China) for development analysis.

**Table 1:** The primers for qRT-PCR

Genes	Forward Primer(5’ to 3’)	Reverse Primer(5’ to 3’)
*Actb*	GTGCTATGTTGCTCTAGACTTCG	ATGCCACAGGATTCCATACC
*Cd36*	CACATACAGAGTTCGTTATCTAGC	CAAAGATGGCTCCATTGGG
*Pparg*	GATGTCTCACAATGCCATCAG	ATATCACTGGAGATCTCCGC
*Egfr*	TGGAGCTATGGTGTCACTG	TGAGATGTCACTTGCTGGG
*Src*	GGCGGTTTCTACATCACCT	GCCATCAGCATGTTTGGAG
*Mmp9*	GTCCAGACCAAGGGTACAG	ATACAGCGGGTACATGAGC
*Esr1*	CCTCTGGCTACCATTATGGG	AGTCATTGTGTCCTTGAATGC

### In vivo experiments

#### Animal models and drug intervention

Eighteen 6-8-week-old male ApoE-/- mice were purchased from Nanjing Junke Biotechnology Co., Ltd. The mice were housed in the laboratory of Cardiology, the 2nd Hospital of Shanxi Medical University, with 6 mice per cage, drinking tap water, and a 12 h photoperiod and housed in an environment with a constant temperature of 25 ℃ and a humidity of 60 ± 10%.

The mice were acclimatized and fed for 7 days before the experiment. Then, 18 mice were randomized to three groups (*n* = 6): the control group (Control), high-fat chow-fed group (HFD) and NOB intraperitoneal injection group (HFD + NOB). The control group received a standard diet, and the other two groups were given a high-fat diet. After an 8-week period of consuming a high-fat diet, the mice received NOB (10 mg/kg) through intraperitoneal injection with once a day for 4 consecutive weeks. Finally, the mice were anesthetized and sacrificed to collect blood, aortic, aortic valve, and carotid artery samples.

#### Oil red O staining

The aortic tissue was removed from 4% paraformaldehyde and rinsed in distilled water. Periarterial fat and connective tissue were removed under a body-view microscope, and the aorta was dissected along its long axis. The mouse aorta was placed in for oil red O staining for 30 min. Then 75% ethanol is added and rinsed until there is no color in normal vessels. After rinsing with distilled water again, the aorta was fixed on a black plate, and the stock image was photographed.

The frozen aortic valves were sectioned, rewarmed and dried, rinsed in distilled water and subsequently dried. Sections were stained with Harris hematoxylin Stain for 5 min, washed in distilled water, and differentiated with differentiation solution. Then, the mixture was returned to blue until the cytoplasm was dark blue and the nucleus was not stained; the cells were subsequently rinsed in running water. Frozen sections were stained with oil red O for 10 min and then rinsed with running water. Finally, the slices were sealed with glycerol gelatin.

#### Hematoxylin-eosin (H&E) staining

Aortic valve tissue was fixed in 10% formalin for 24 h at cold temperature (4 °C) and encased in paraffin. Then, 5 μm sections were prepared to stain with H&E according to standard routine protocols.

#### Lipid testing

The working solution of each reagent was prepared, the parameters of the automatic biochemical analyzer were set, serum specimens were sampled, and the automatic biochemical analyzer automatically determined the lipid levels of each group of mice, namely, TC and TG.

#### Immunofluorescence (IF) staining

IF was performed for CD36 and CD68. Cryosections were fixed in ice-cold methanol for 10 min and washed in PBS, and nonspecific staining was blocked by incubation with 3% BSA. Staining for CD36: rabbit anti-CD36 (Servicebio, Wuhan, China) and FITC-conjugated goat anti-rabbit (Servicebio, Wuhan, China). Staining for CD68: mouse anti-CD68 (Santa Cruz Biotechnology, Northern California, USA) and Cy3-conjugated donkey anti-mouse (Servicebio, Wuhan, China). Ultimately, the sections were contrast-stained with DAPI (Boster Biotech, Wuhan, China) to visualize the nuclei. A Leica TCSSP8 DMI8 LASX microscope with Leica LASX software was used for imaging.

### Statistical analysis

During the data extraction and analysis, the following software was applied: R software 4.1.0, Strawberry Perl software 5.30.1–64, and GraphPad Prism software 8.0.2. In the analyses of the experimental data, the variations among multiple groups were analyzed using one-way ANOVA, followed by a Tukey post hoc test. Statistical significance is indicated by a P value lower than 0.05. The data are shown as the mean ± SD from at least three independent experiments. The data were obtained from at least three independent experiments per group.

## Results

### A total of 141 genes were identified as potential targets through which NOB could intervene in atherosclerosis

**Fig. 2:**
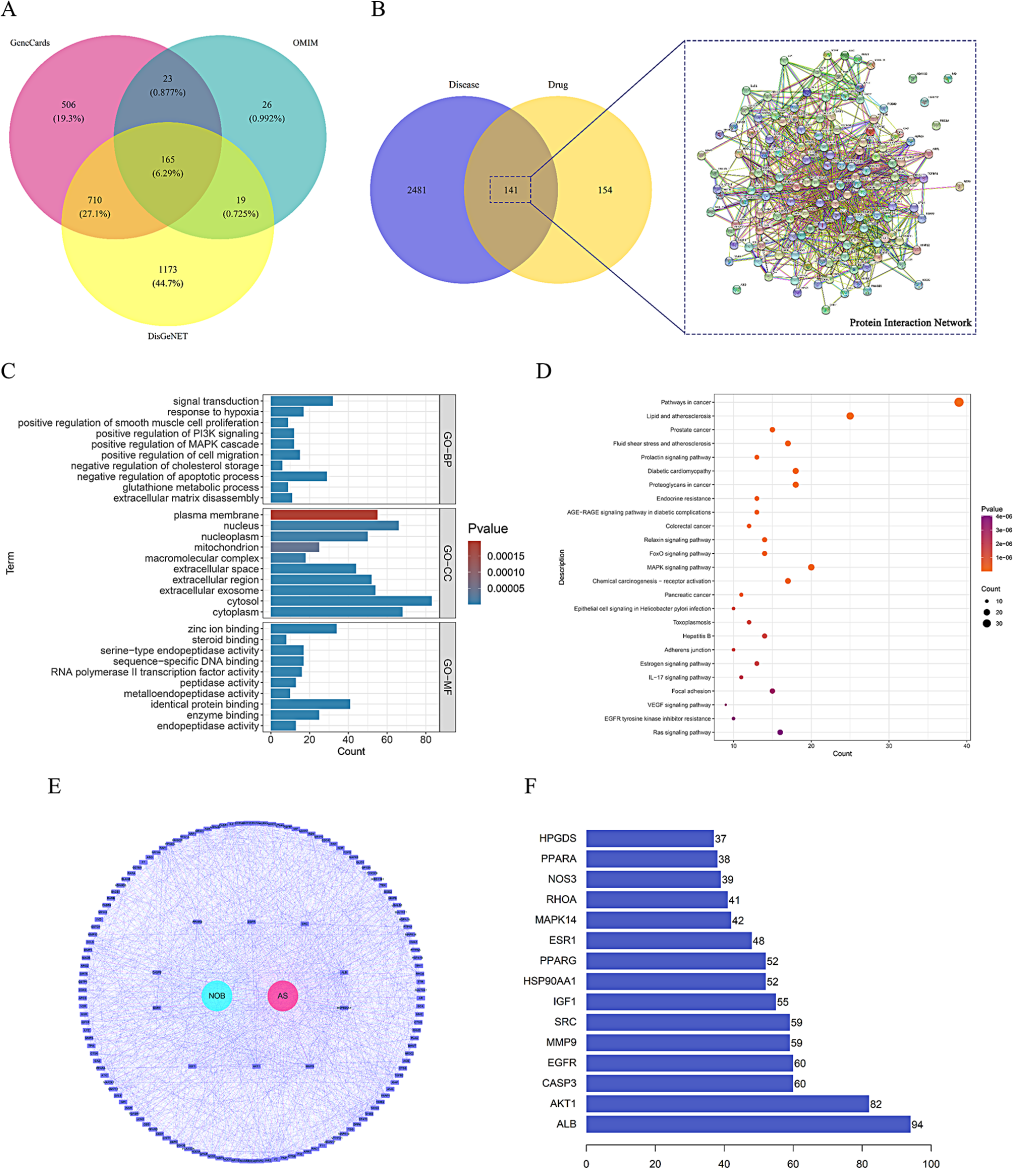
(**A**) Contribution of three databases to atherosclerotic disease-relevant targets. (**B**) On the left is a Venn diagram of NOB binding targets and atherosclerotic disease-associated targets. The PPI network constructed from STRING is shown on the right. (**C**) Based on the GO enrichment analysis, functions in which NOB targets may be involved in atherosclerosis, including biological processes, cellular components and molecular functions, were identified. (**D**) KEGG analysis results showing pivotal signaling pathways associated with NOB intervention in atherosclerosis. The dimension of the bubbles shows the number of genes, and the color stands for the *P*-value. (**E**) A network of NOB intervention in atherosclerosis. (**F**) The top 10 core targets through which NOB may intervene in atherosclerosis according to the degree method are depicted in the bar plot

The PharmMapper server produced 295 NOB targets, and the three chosen databases provided 2622 atherosclerosis targets. The distribution of atherosclerosis targets in the three databases is depicted in Fig. 2A. Overall, 141 targets were identified through which NOB might intervene in atherosclerosis (Fig. 2B). The removal of reread targets during target screening is common practice.

### Lipids and atherosclerosis may be the dominant pathways and potential underlying mechanisms

Based on the GO enrichment analysis, NOB intervention in atherosclerosis mainly involved negative regulation of the apoptotic process, positive regulation of PI3K signaling, and positive regulation of smooth muscle cell proliferation (Fig. 2C). According to KEGG enrichment analysis, NOB interferes with atherosclerosis primarily through lipids and atherosclerosis, fluid shear stress and atherosclerosis signaling pathways (Fig. 2D). Important molecular mechanisms involved in lipid metabolism and atherosclerosis are shown in Supplementary Figs. 1 and 2.

### Protein network construction and determination of important connections

PPIs were obtained from STRING, and the network of interactions between NOB and atherosclerosis was plotted via the application of Cytoscape. This network was constructed as a map of 141 targets related to NOB intervention in atherosclerosis patients (Fig. 2E). The magenta dot in the center represents the disease atherosclerosis, and the blue dot represents the drug NOB. The surrounding purple squares represent 141 targets. In addition, the connecting lines also follow the color rule. These 141 targets were uploaded into Cytoscape software and ranked using topological analysis methods (Degree, MCC, MNC, EPC, Closeness and Betweenness) provided by the CytoHubba plugin, and the core targets are shown in Table 2. Despite slight differences in ranking, the following genes were among the top ten genes without controversy: ALB, AKT1, CASP3, EGFR, SRC, MMP9, IGF1, HSP90AA1, PPARG, and ESR1. The ranking of each target gene can be seen in Fig. 2F according to the degree method.

**Table 2:** Core targets calculated based on multiple algorithms

Category	Rank methods in CytoHubba					
	Degree	MCC	MNC	EPC	Closeness	Betweenness
1	ALB	CASP3	ALB	AKT1	ALB	ALB
2	AKT1	SRC	AKT1	ALB	AKT1	AKT1
3	CASP3	HSP90AA1	CASP3	IGF1	EGFR	EGFR
4	EGFR	ALB	SRC	SRC	CASP3	PPARG
5	SRC	EGFR	MMP9	CASP3	SRC	CASP3
6	MMP9	IGF1	EGFR	EGFR	MMP9	SRC
7	IGF1	AKT1	IGF1	MMP9	IGF1	MMP9
8	HSP90AA1	MMP9	PPARG	HSP90AA1	PPARG	G6PD
9	PPARG	MAPK14	HSP90AA1	PPARG	HSP90AA1	HSP90AA1
10	ESR1	MAPK8	ESR1	ESR1	ESR1	ESR1

*MCC*: maximal clique centrality, *MNC*: maximum neighborhood component, *EPC*: edge percolated component

**Table 3:** Binding affinity calculation of NOB and its core targets

Targets	PDB ID	Affinity(kcal/mol)	AA
ALB	1E7A	-8.102	ALA, GLN, HIS, LYS, SER
AKT1	7NH5	-8.668	TRP, TYR, ASN
CASP3	6X8I	-7.677	PRO, VAL, ARG
MMP9	6ESM	-7.927	VAL, HIS, TYR, LEU, GLN
EGFR	7JXQ	-7.293	VAL, ALA, PHE, LYS
SRC	1O43	-5.916	ALA, TYR, ASN, HIS, PHE
IGF1	1IMX	-5.457	LEU
HSP90AA1	7S9H	-7.685	LEU, PHE
PPARG	7E0A	-9.692	ARG, ILE
ESR1	7RS8	-7.230	ALA, TRP, LEU

### NOB strongly binds to core targets

**Fig. 3:**
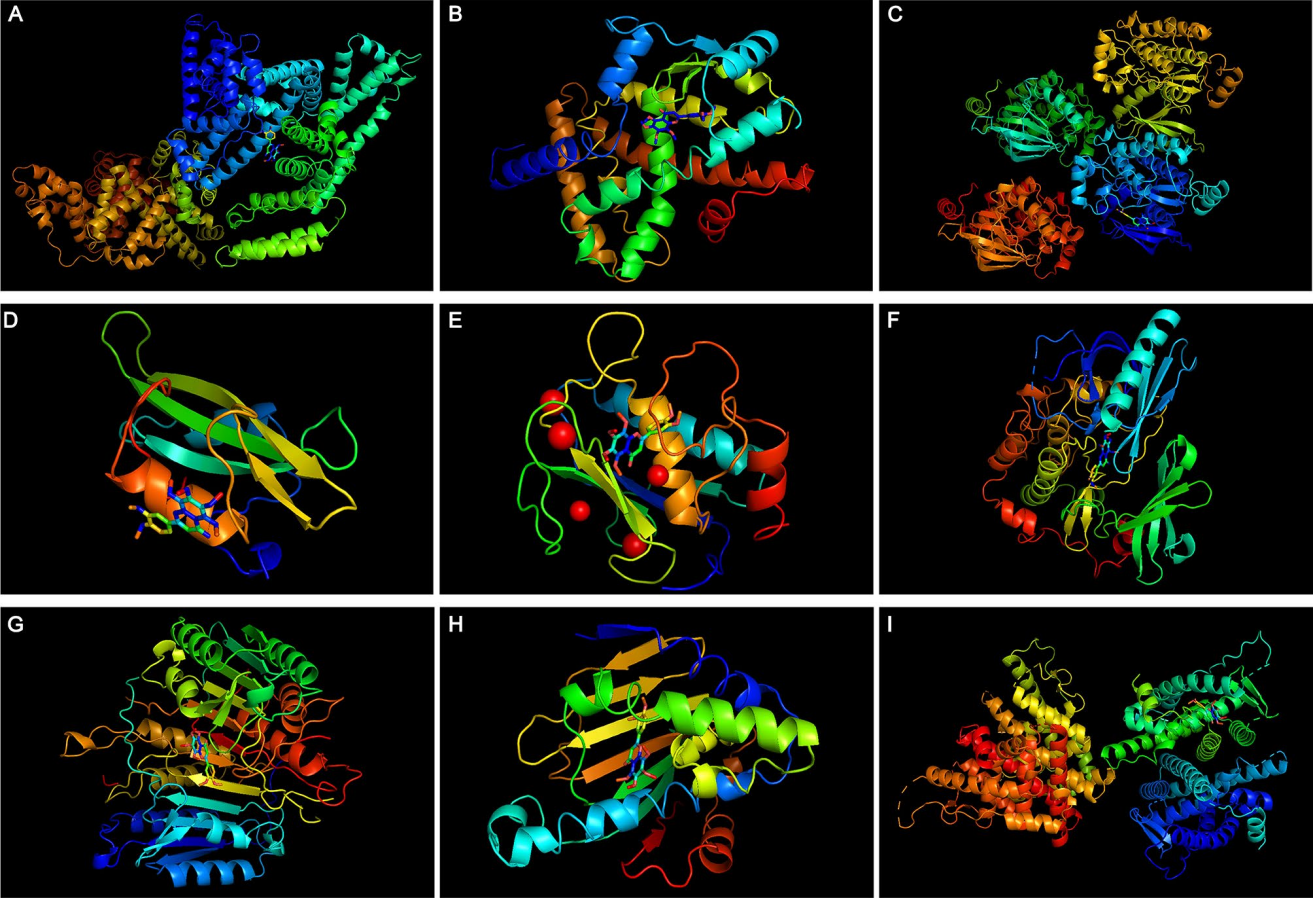
Overall display of docking results. The protein receptor (PDB ID) sequences in Figure (**A-I**) were 1E7A, 7E0A, 7JXQ, 1O43, 6ESM, 7NH5, 6X8I, 7S9H, and 7RS8

**Fig. 4:**
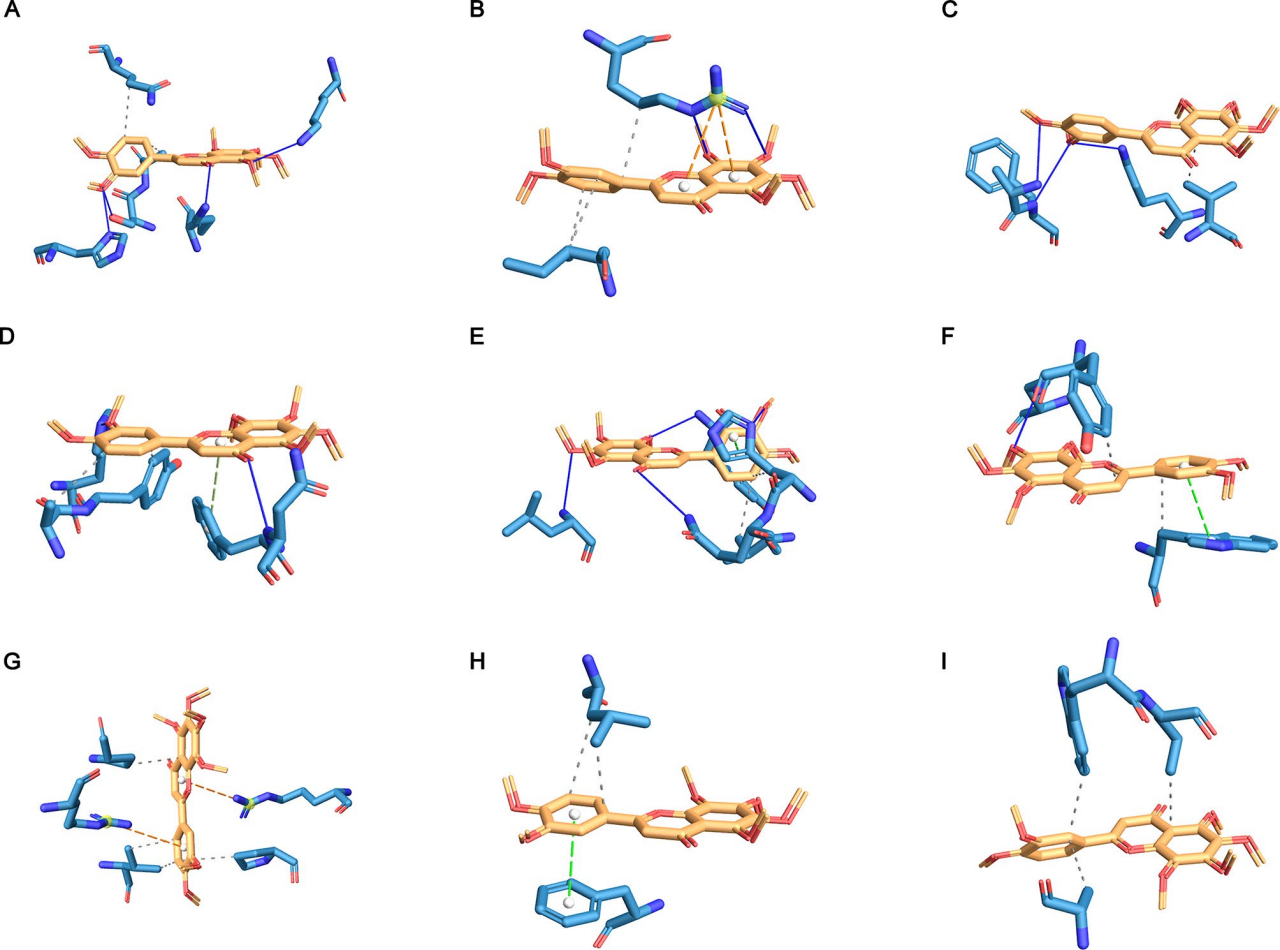
Mutual binding between protein receptors and ligands. The PDB IDs in Figure (**A-I**) were 1E7A, 7E0A, 7JXQ, 1O43, 6ESM, 7NH5, 6X8I, 7S9H, and 7RS8

Molecular docking simulation analyses were also conducted on the top ten core targets. Table 3 showed that these NOBs bound steadily to the core targets (affinity > 5 kcal/mol), validating CytoHubba’s calculations. NOB docked with core targets in PyMOL, as depicted in Fig. 3. Figure 4 shows the analysis of noncovalent interactions with the application of PLIP. The purple straight line represents the hydrogen bond. The gray, light green, dark green, and orange dotted lines represent hydrophobic interactions, π-stacking (parallel), π-stacking (perpendicular), and π-cation interactions, respectively.

### Six genes related to the M0 macrophage fraction

The targets of NOB were enriched mainly in the lipids and atherosclerosis signal pathway, while in the GO-BP, there was also a role in the regulation of cholesterol storage (Fig. 2C, Supplementary Fig. 1). In addition, PPARG plays an important role in lipid uptake by macrophages. Therefore, this work focused on the role of NOB in inhibiting macrophage lipid uptake through the PPARG/CD36 pathway.

**Fig. 5:**
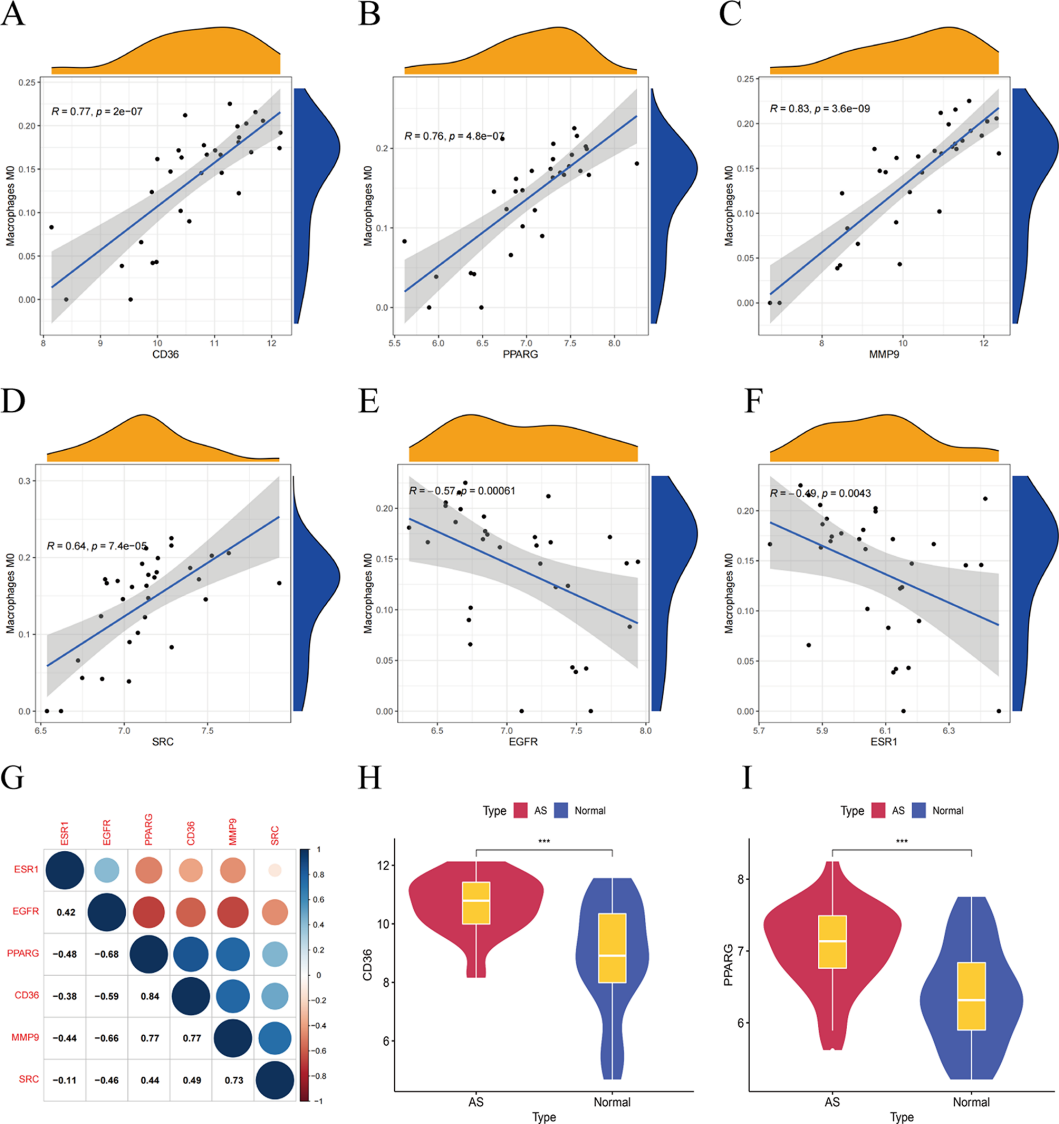
The correlations between the expression of the six genes (PPARG, MMP9, SRC, CD36, EGFR and ESR1) and the M0 macrophage fraction are depicted in the Figures (**A-F**). (**G**) Correlation analysis of the six genes associated with M0 macrophages. (**H-I**) Analysis of differences between PPARG and CD36 in normal and atherosclerotic samples

Immune cell infiltration analysis and related gene expression analysis were performed on 32 carotid atherosclerotic plaque tissues and 32 adjacent normal carotid tissues. Spearman’s method was used to analyze the correlation between immune cell infiltration scores and related gene expression in atherosclerotic plaques. It showed that the mRNA levels of PPARG, MMP9, SRC and CD36 were positively correlated with the M0 macrophage fraction (Fig. 5A-D). The mRNA levels of EGFR and ESR1 was negatively correlated with the M0 macrophage fraction (Fig. 5E, F). In atherosclerosis, PPARG promoted the differentiation of peripheral blood monocytes to tissue macrophages and macrophage infiltration [53]. While CD36 is associated with macrophage lipid uptake, inflammatory response, and also recruits macrophages [54]. MMP9 degrades the extracellular matrix to promote inflammatory cell infiltration, migration, and then disrupts the normal structure of tissues by acting in concert with inflammatory factors [55]. SRC is associated with lipid uptake by macrophages [56]. In addition, it has been shown that activation of EGFR and ESR1 has an inhibitory effect on macrophage migration [57, 58]. When PPARG expression was continuously upregulated, CD36 expression was also increased, and both were positively correlated (Fig. 5G). Among them, CD36 and PPARG gene expression differed between normal samples (*n* = 32) and atherosclerotic samples (*n* = 32) (Fig. 5H, I).

### NOB inhibited lipid uptake by RAW264.7 cells and thus prevented foam cell formation

**Fig. 6:**
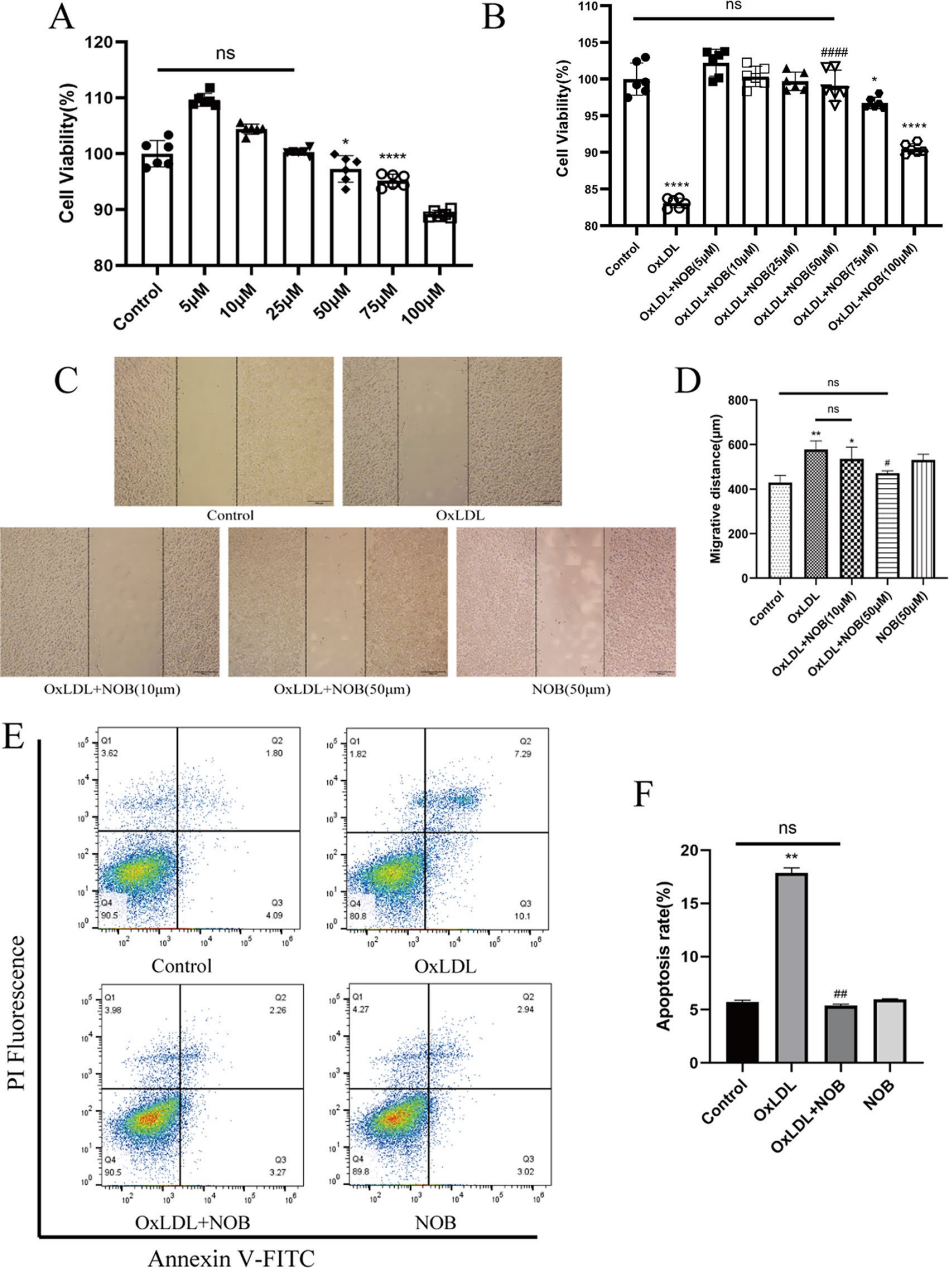
Influence of NOB on RAW264.7 cells viability. RAW264.7 cells were intervened by OxLDL (50 µg/mL) for 48 h to construct an ex vivo model of atherosclerosis. (**A**) The direct effect of different concentrations of NOB on RAW264.7 cell viability was analyzed by CCK-8 method (*n* = 6). (**B**) On the basis of an ex vivo model of atherosclerosis, CCK-8 was used to detect the ameliorative effect of NOB on cell viability (*n* = 6). The effect of OxLDL on macrophage migratory capacity (at six random locations; 200× magnification) and the ameliorative effect of NOB were observed via a scratch assay (**C, D**) (*n* = 6). The ameliorative effect of NOB (50 µM) on OxLDL-induced apoptosis was examined using flow cytometry (**E, F**) (*n* = 3). *P < 0.05, **P < 0.01, ****P < 0.0001 vs. the control group; #*P* < 0.05, ##*P* < 0.01, ####*P* < 0.0001 vs. the OxLDL group

First, the effects of different levels of NOB on RAW264.7 cells viability were examined. The concentrations of 5–25 µM of NOB failed to inhibit RAW264.7 cell viability significantly, while the concentration of 50 µM of NOB began to inhibit cell viability (Fig. 6A). To elucidate the anti-atherosclerotic pharmacological effects of NOB, we treated RAW264.7 cells with OxLDL (50 µg/mL) as an ex vivo model of atherosclerosis. CCK-8 analysis revealed that OxLDL significantly reduced cell survival, while 50 µM NOB significantly increased cell viability (Fig. 6B). Therefore, a 50 µM concentration of NOB was selected for the subsequent experiments. According to the scratch assay, OxLDL significantly inhibited the migration of RAW264.7 cells, while NOB reversed this effect (Fig. 6C, D). Apoptosis analysis showed that OxLDL-mediated apoptosis could be ameliorated by NOB. In conclusion, NOB protects RAW264.7 cells from OxLDL-induced viability inhibition and apoptosis (Fig. 6E, F).

### Trends of NOB action on target genes in an ex vivo model of atherosclerosis

A model of atherosclerosis was constructed, and NOB intervention was performed. The qRT‒PCR was used to confirm the influence of NOB on target genes. In atherosclerosis, the expression levels of CD36, PPARG, MMP9 and SRC were upregulated, while NOB inhibited their expression (Supplementary Fig. 3A-D). The expression of EGFR and ESR1 was reduced, and NOB had the opposite effect (Supplementary Fig. 3E, F). Thus, these six target genes are associated with macrophages in atherosclerosis and are targets of NOB regulation.

### NOB inhibits lipid uptake in macrophages by modulating the PPARG signaling pathway

**Fig. 7:**
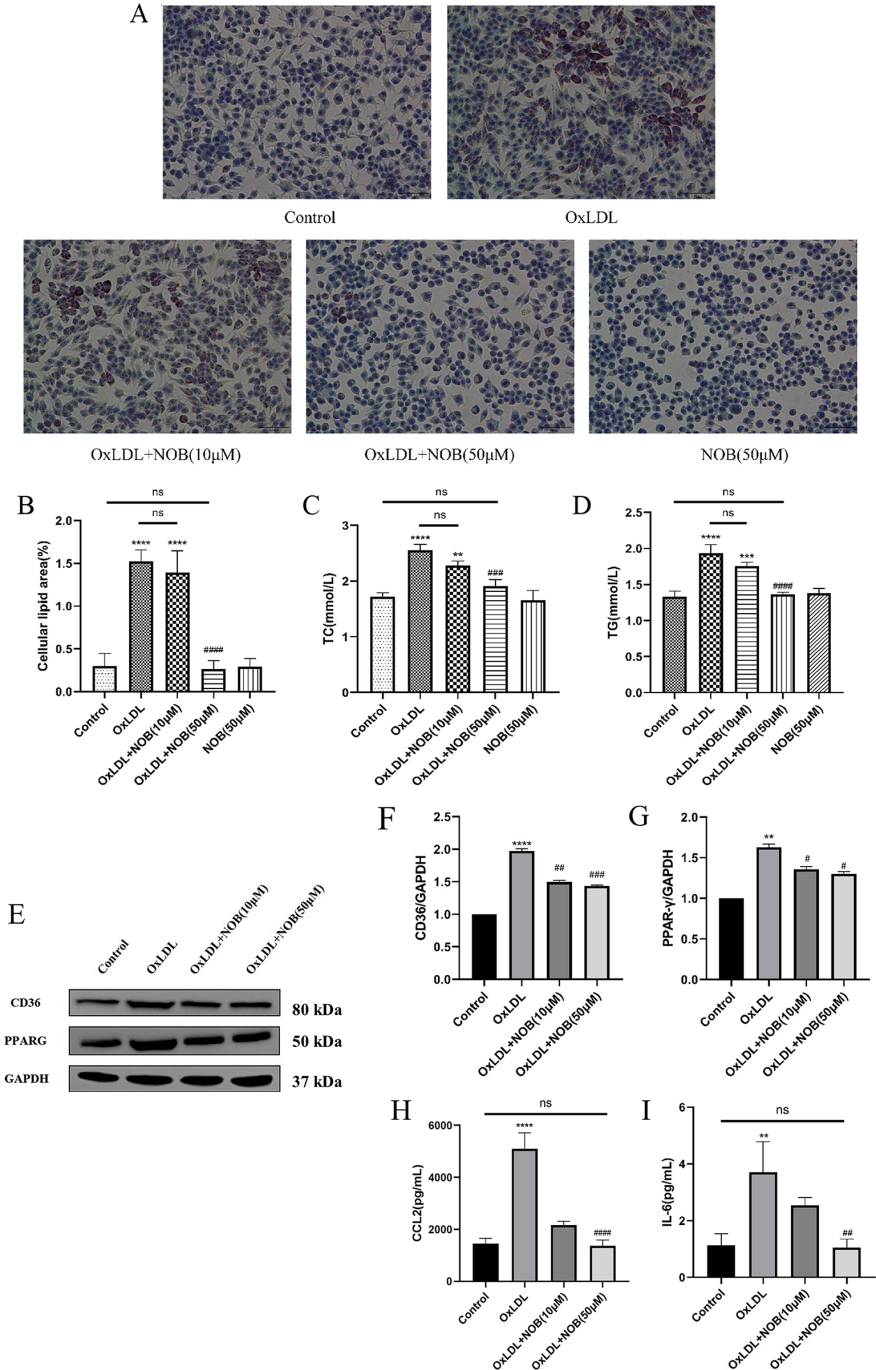
By modulating the PPARG/CD36 signaling pathway, NOB inhibits foam cells formation. An ex vivo model of atherosclerosis was constructed, and the model was deintervened with NOB (10 µM, 50 µM) for 48 h. RAW264.7 Lipid droplet content in cells is tested using Oil Red O staining (at six random locations; 400× magnification) (**A, B**) (*n* = 6). The intracellular expression levels of TC and TG were measured by biochemical kits according to the above grouping and interventions (**C, D**) (*n* = 3). Different concentrations of NOB were used to treat an ex vivo model of atherosclerosis, and the protein content of CD36 and PPARG was measured by WB (**E-G**) (*n* = 3). Inflammatory factors CCL2 and IL-6 in cell supernatants were quantified by ELISA (**H, I**) (*n* = 3). **P < 0.01, ***P < 0.001, ****P < 0.0001 vs. the control group; #*P* < 0.05, ##*P* < 0.01, ###*P* < 0.001, ####*P* < 0.0001 vs. the OxLDL group

Subsequently, it was further elucidated whether NOB could hinder the creation of foam cells by manipulating the PPARG signaling pathway. Oil red O staining of the cells revealed that OxLDL significantly promoted foam cell formation, while NOB dose-dependently reduced the number of lipid droplets in macrophages (Fig. 7A, B). After the cell homogenates were assayed for total cholesterol (TC) and triglyceride (TG) levels, NOB was found to inhibit lipid phagocytosis by macrophages (Fig. 7C, D). OxLDL induced the expression of the PPARG/CD36 mRNAs and proteins, and these effects were reversed by NOB intervention (Supplementary Fig. 3A, B; Fig. 7E-G). Therefore, NOB reduces OxLDL-induced foam cells formation by regulating the PPARG signaling pathway.

Additionally, the results showed that NOB has anti-inflammatory effects. After OxLDL intervention, RAW264.7 cells released inflammatory factors (CCL2 and IL6), and NOB inhibited their expression (Fig. 7H, I).

### NOB attenuates atherosclerotic lesions in mice in vivo

**Fig. 8:**
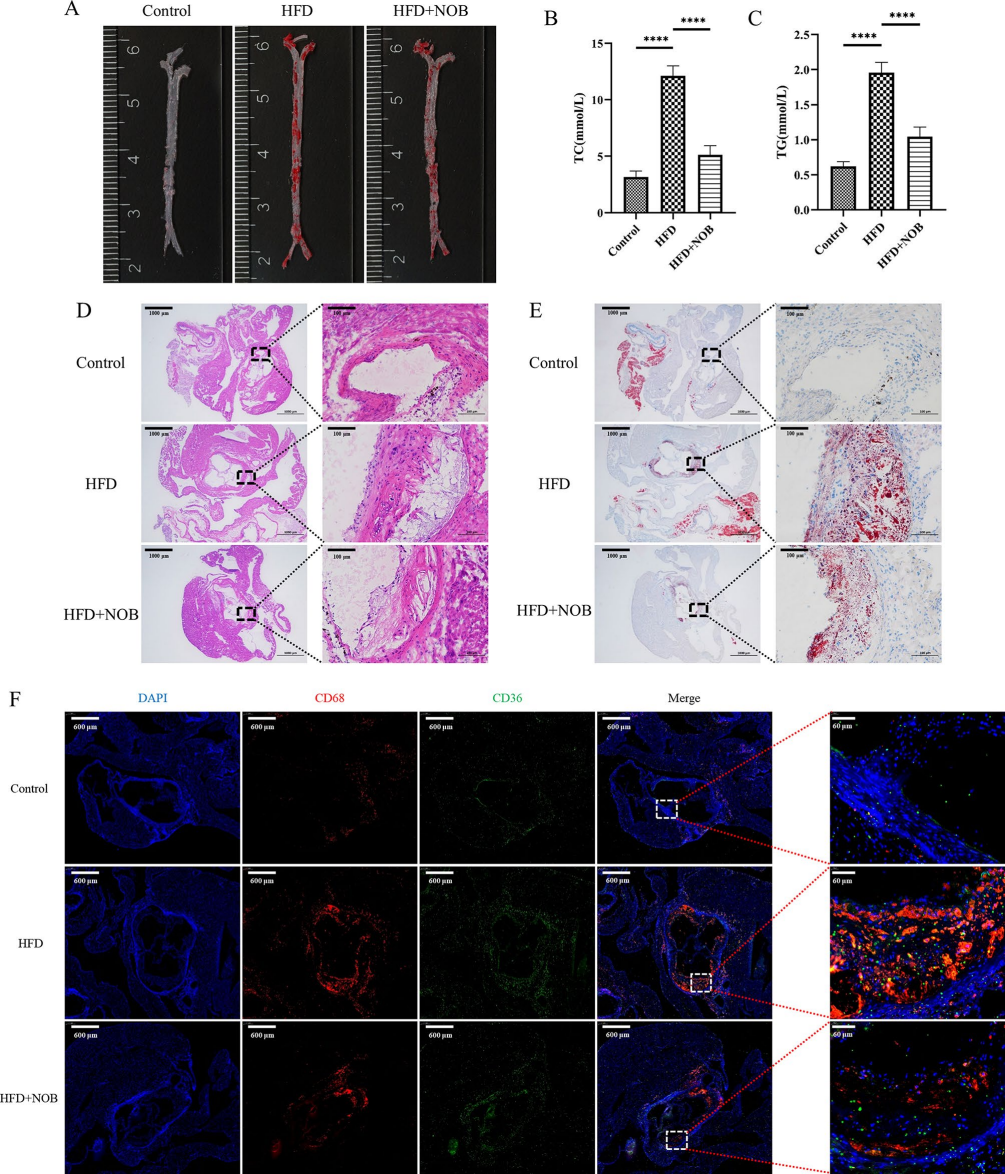
(**A**) The general oil red O staining of mouse aortas. (**B, C**) The TC and TG analysis of mouse serum (*n* = 3). (**D, E**) H&E staining and oil red O staining of mouse aortic valves (1000 μm, 100 μm). (**F**) Immunofluorescence colocalization of CD68 (red) and CD36 (green) in mouse aortic valves (600 μm, 60 μm). ****P < 0.0001

Oil red O staining of the whole aortas of the mice revealed that NOB attenuated lipid accumulation in the aorta (Fig. 8A). Biochemical testing of mouse serum showed that NOB effectively reduced TG and TC levels in mice (Fig. 8B, C). H&E staining and oil red O staining of aortic valves displayed that NOB reduced atherosclerotic plaque production and lipid accumulation (Fig. 8D, E). Similarly, macrophage infiltration was reduced, and CD36 expression was decreased in the aortic valve and carotid artery of mice in the NOB intervention group (Fig. 8F; Supplementary Fig. 4).

## Discussion

Atherosclerotic vascular disease is the leading cause of global human mortality [59]. The main risk factors for atherosclerosis are hyperlipidemia, diabetes mellitus, smoking and hypertension [60]. Atherosclerosis is a chronic inflammatory disease, and foam cells are the typical pathological cells that form mainly due to phagocytosis of lipids by macrophages and vascular smooth muscle cells (VSMC) [61, 62]. Among them, CD36 and scavenger receptor class A (SR-A) are key receptors for OxLDL uptake by macrophages and promote foam cells formation [63, 64]. Moreover, macrophage accumulation also promotes inflammatory effects and accelerates disease progression [65]. Macrophages are crucial in the development and rupture of atherosclerotic plaques and serve as possible targets for therapy [66–68].

With the application of network pharmacology, many herbs that are effective against atherosclerosis have been reported [69, 70]. NOB is the main active constituent of citrus fruits and has rich pharmacological, antitumor, antiviral, anti-inflammatory, antioxidant and antidiabetic effects [22, 71, 72]. Stewart et al. reported that NOB, a citrus flavonoid isolated from orange, selectively inhibited the SR-A receptor-mediated metabolism of acetylated low-density lipoproteins in mouse macrophages [73]. Mulvihill et al. reported that NOB increased insulin sensitivity and glucose tolerance in the liver and circulation of mice and significantly attenuated aortic sinusoidal atherosclerosis [23]. In addition, in a recent review, it was reported that a preclinical evaluation confirmed the regulatory influence of flavonoids on adhesion molecules in atherosclerosis [74]. However, the mechanism of effects of NOB in ameliorating atherosclerosis is not fully understood.

In this research, it has been explored how NOB improved atherosclerosis for the first time using network pharmacology, molecular docking simulation techniques, immune cell prediction analysis and an atherosclerotic cell model. Altogether, 141 potentially targetable genes were identified through which NOB could intervene in atherosclerosis based on three public databases. STRING and CYTOSCAPE was applied to visually analyze the PPI network of these genes, ALB, AKT1, CASP3, EGFR, SRC, MMP9, IGF1, HSP90AA1, PPARG, and ESR1 were identified as the top 10 key targets. This finding was verified by molecular docking. According to immune-related analysis of an external dataset (GSE43292), the immune cell fraction was predicted in 32 clinical samples of atherosclerotic plaques, and a correlation was found between M0 macrophages and the expression of key targets (PPARG and CD36). Moreover, OxLDL-treated macrophages are widely used as an ex vivo model of atherosclerosis. Therefore, this study focused mainly on how NOB improves atherosclerosis by targeting OxLDL-treated macrophages.

**Fig. 9:**
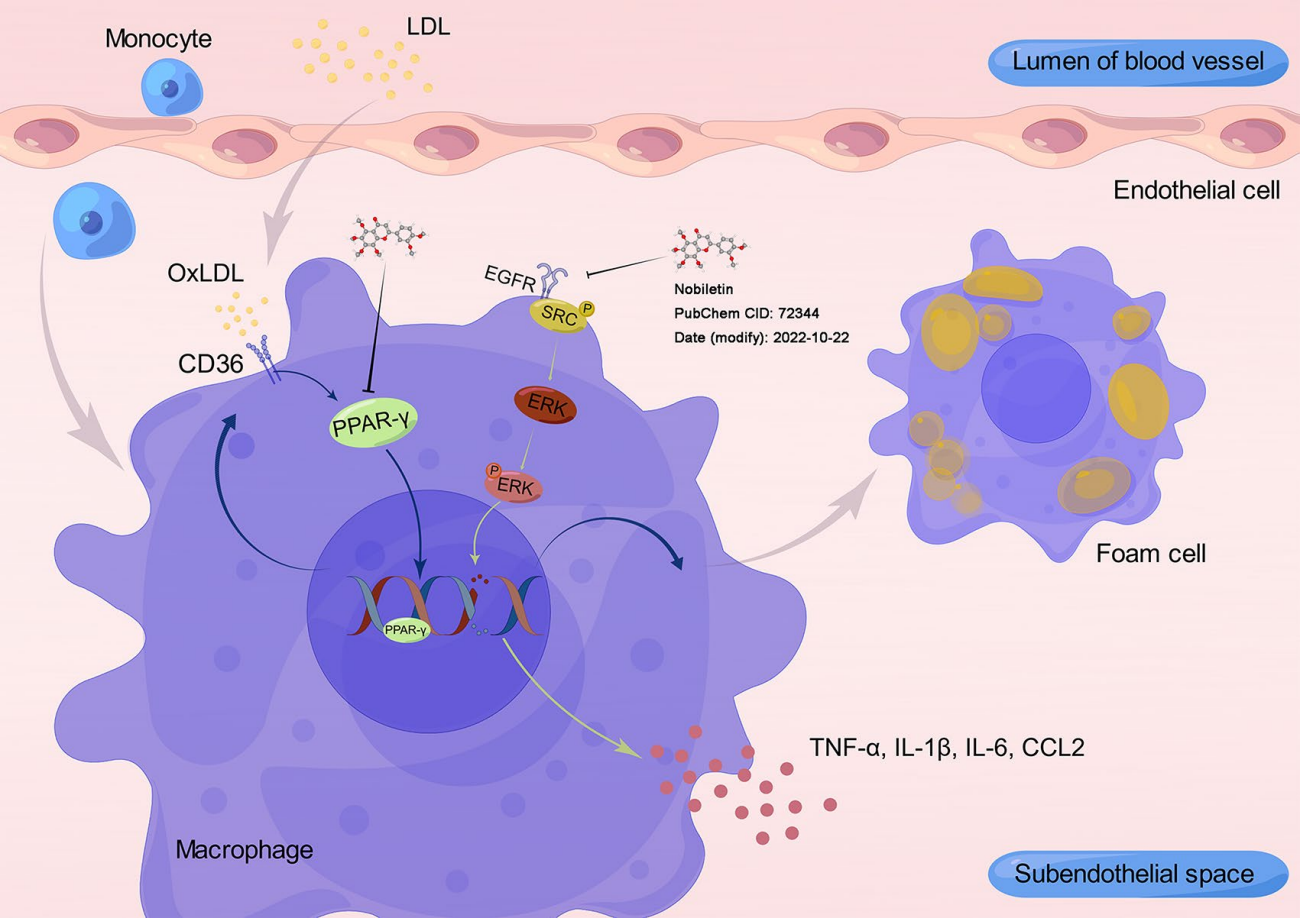
This is a new working model for illustrating the protective mechanism of NOB in atherosclerosis. OxLDL is transported into macrophages via CD36 to produce fatty acid derivatives that activate PPARG and promote its expression. PPARG is a transcription factor that regulates the expression of CD36, which in turn promotes lipid uptake. NOB inhibits the formation of foam cells and in addition exerts an anti-inflammatory effect

PPAR-α, PPAR-δ and PPARG are transcription factors that regulate gene expression following ligand activation [75]. For diseases such as atherosclerosis, inflammation and hypertension, PPAR is considered an important therapeutic target [76]; among them, PPARG plays an important role in regulating atherosclerosis. OxLDL enters macrophages and activates PPARG by providing oxidized fatty acids, thereby enhancing the expression of proteins such as CD36 and nuclear liver X receptor (LXRα) [77, 78]. CD36 has the powerful ability to capture OxLDL and is considered to be a key component of foam cells formation and a major pro-atherosclerotic factor [79–81]. PPARG controls the transcription of many genes controlling lipid metabolism by PPARG binds mono- and polyunsaturated fatty acids and their derivatives (e.g., arachidonoids) in different ways to control the transcription of many genes that control lipid metabolism [82]. PPARG is less responsive to natural fatty acids than other PPARs, and oxidized fatty acid derivatives contained in circulating oxidized low-density lipoprotein (oxLDL) cause strong activation of PPARG. At the molecular level, internalized oxLDL provides oxidized fatty acids as ligands for PPARγ, the major transcription factor for CD36, which activates CD36 gene expression to further increase oxLDL uptake. In addition, Nagy et al. [83] demonstrated that as a consequence of oxLDL internalization by CD36-mediated endocytosis, an activating signal for PPARγ is forwarded to the nucleus. Transcriptional activation of PPARγ initiates a positive feedback loop that enhances the expression of the oxLDL receptor CD36. And in their follow-up study [53], they confirmed that the CD36 gene promoter is a direct target of PPARG, which activates the transcription of the CD36 gene in a ligand-dependent manner. Interestingly, it has been reported in the literature that activation of PPARG simultaneously increases SR-B1 and ABC transporter protein-dependent cholesterol efflux, which in turn reduces foam cell formation [84–86]. In addition, regulating the PPARG signaling pathway also inhibits inflammation and stabilizes atherosclerotic plaques [85, 87, 88]. The underlying mechanism is illustrated in Fig. 9.

Consistent with our expectations, ex vivo experiments on atherosclerosis have demonstrated that NOB can interfere with the atherosclerotic process by modulating core targets, such as ALB, AKT1, and CASP3. This study aimed to explore the molecular mechanisms by which NOB ameliorates atherosclerosis by regulating macrophages. The experimental results showed that NOB inhibited CD36-mediated lipid uptake by macrophages by modulating the PPARG signaling pathway, further reducing foam cell formation. Since foam cells are crucial in the early development of atherosclerosis and in the progressive rupture phase, this ameliorating effect deserves to be studied in depth in the future.

## Strengths and limitations

This research has many strengths. The first is to elucidate the ameliorative effect of NOB on atherosclerosis through network pharmacology analysis and cell and animal experiments. NOB can inhibit lipid uptake by macrophages by modulating the PPARG/CD36 signaling pathway. In addition, since NOB is the main natural active ingredient of citrus fruits, NOB may be useful for preventing atherosclerosis-like cardiovascular diseases.

This study also has several limitations. This research focused on improving atherosclerosis by modulating the PPARG/CD36 pathway in macrophages. In fact, the PPARG/CD36 signaling pathway is crucial for various cell types, including vascular endothelial cells (VEC), VSMC, and T cells. A wider range of cell types should be included in future research. 2) Notably, the ERK1/2 signaling pathway also contributes significantly to atherosclerosis. Activity of the EGFR/SRC/ERK1/2 pathway is closely associated with inflammatory responses, macrophage polarization and foam cell formation. NOB may be able to perform anti-inflammatory action by regulating the ERK1/2 signaling pathway, which will be explored in subsequent studies. Furthermore, in cellular experiments, the regulatory effect of NOB on reducing PPARG/CD36 protein expression was limited. However, the exact reason for this is unclear. In future studies, it will be necessary to continue to explore how NOB is functionally regulated after binding to its targets.

## Conclusion

In summary, network pharmacology, bioinformatics methods and ex vivo and in vivo experimental validation were applied to reveal NOB’s action against atherosclerosis at the molecular level. NOB inhibits lipid uptake by macrophages mainly by modulating the PPARG/CD36 signaling pathway, which in turn exerts antiatherosclerotic effects. Drugs targeting the PPARG/CD36 signaling pathway are promising therapeutic options for the therapy of atherosclerosis. NOB is expected to be employed as an active natural component for preventing and treating atherosclerotic disease. Since NOB is the main active ingredient of citrus fruits, consuming a moderate amount of citrus fruits in daily life has the effect of protecting blood vessels against atherosclerosis.

### Electronic supplementary material

Below is the link to the electronic supplementary material.

Supplementary Material 1

Supplementary Material 2

Supplementary Material 3

Supplementary Material 4

Supplementary Material 5

## Data Availability

The expression profiles of clinical samples are available in the GEO database. The original contributions presented in the study are included in the article/supplementary material. Further inquiries can be directed to the corresponding authors.

## Abbreviations

AS: Atherosclerosis
NOB: Nobiletin
OxLDL: Oxidized low-density lipoprotein
PPARG/PPAR-γ: Peroxisome proliferator-activated receptor-γ
PPI: Protein‒protein interaction
MNC: Maximum Neighborhood Component
EPC: Edge Percolated Component
MCC: Maximal Clique Centrality
PDB: Protein Data Bank
FBS: Fetal bovine serum
CCK-8: Cell Counting Kit-8
PBS: Phosphate-buffered saline
TC: Total cholesterol
TG: Triglycerides
qRT‒PCR: Quantitative real-time PCR
WB: Western blot
PVDF: Polyvinylidene fluoride
H&E: Hematoxylin-eosin
IF: Immunofluorescence
SR-A: Scavenger receptor class A
LXRα: Liver X receptor
