# Supplementary material for: Nobiletin alleviates atherosclerosis by inhibiting lipid uptake via the PPARG/CD36 pathway
Source: Lipids Health Dis. 2024 Mar 11;23:76. doi: 10.1186/s12944-024-02049-5 (PMC10926578; doi:10.1186/s12944-024-02049-5)
Supplement: Supplementary file 1 — Supplementary Material 1 [file 12944_2024_2049_MOESM1_ESM.pdf]

# Editing Certificate

This document certifies that the manuscript

**Nobiletin alleviates atherosclerosis by inhibiting lipid uptake via the PPARG/CD36 pathway**

prepared by the authors

**Heng Wang, Qinqin Tian, Ruijing Zhang, Qiujing Du, Jie Hu, Tingting Gao, Siqi Gao, Keyi Fan, Xing Cheng, Sheng Yan, Guoping Zheng, Honglin Dong**

was edited for proper English language, grammar, punctuation, spelling, and overall style by one or more of the highly qualified native English speaking editors at AJE.

This certificate was issued on **January 22, 2024** and may be verified on the AJE website using the verification code **F289-5C19-47C3-51EE-8EC5**.

Neither the research content nor the authors' intentions were altered in any way during the editing process. Documents receiving this certification should be English-ready for publication; however, the author has the ability to accept or reject our suggestions and changes. To verify the final AJE edited version, please visit our verification page at aje.com/certificate. If you have any questions or concerns about this edited document, please contact AJE at support@aje.com.

AJE provides a range of editing, translation, and manuscript services for researchers and publishers around the world.
For more information about our company, services, and partner discounts, please visit aje.com.
