# Supplementary figures and images for: Nobiletin alleviates atherosclerosis by inhibiting lipid uptake via the PPARG/CD36 pathway
Source: Lipids Health Dis. 2024 Mar 11;23:76. doi: 10.1186/s12944-024-02049-5 (PMC10926578; doi:10.1186/s12944-024-02049-5)

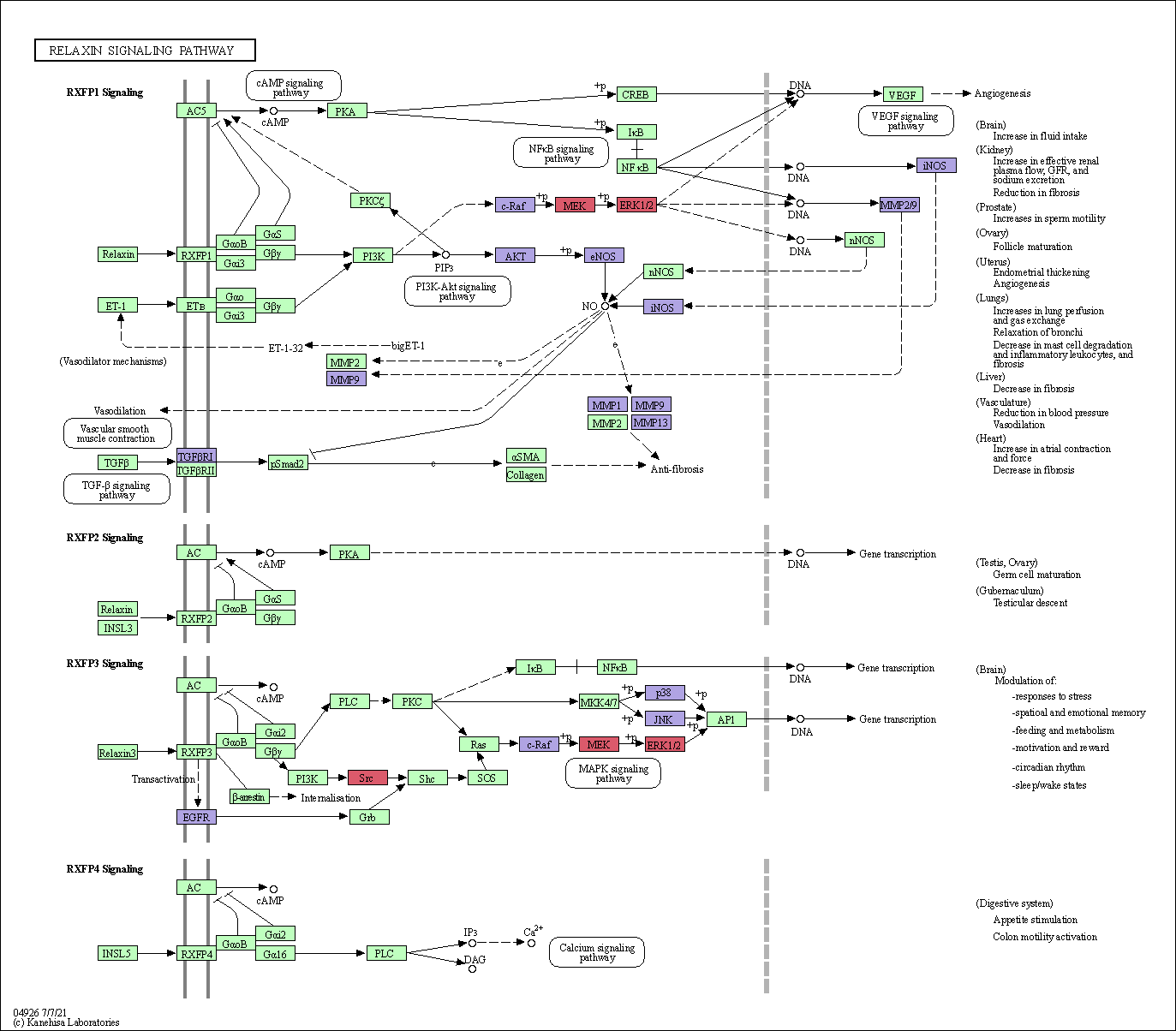

Supplement: Supplementary file 2 — Supplementary Material 2 [file 12944_2024_2049_MOESM2_ESM.tif]

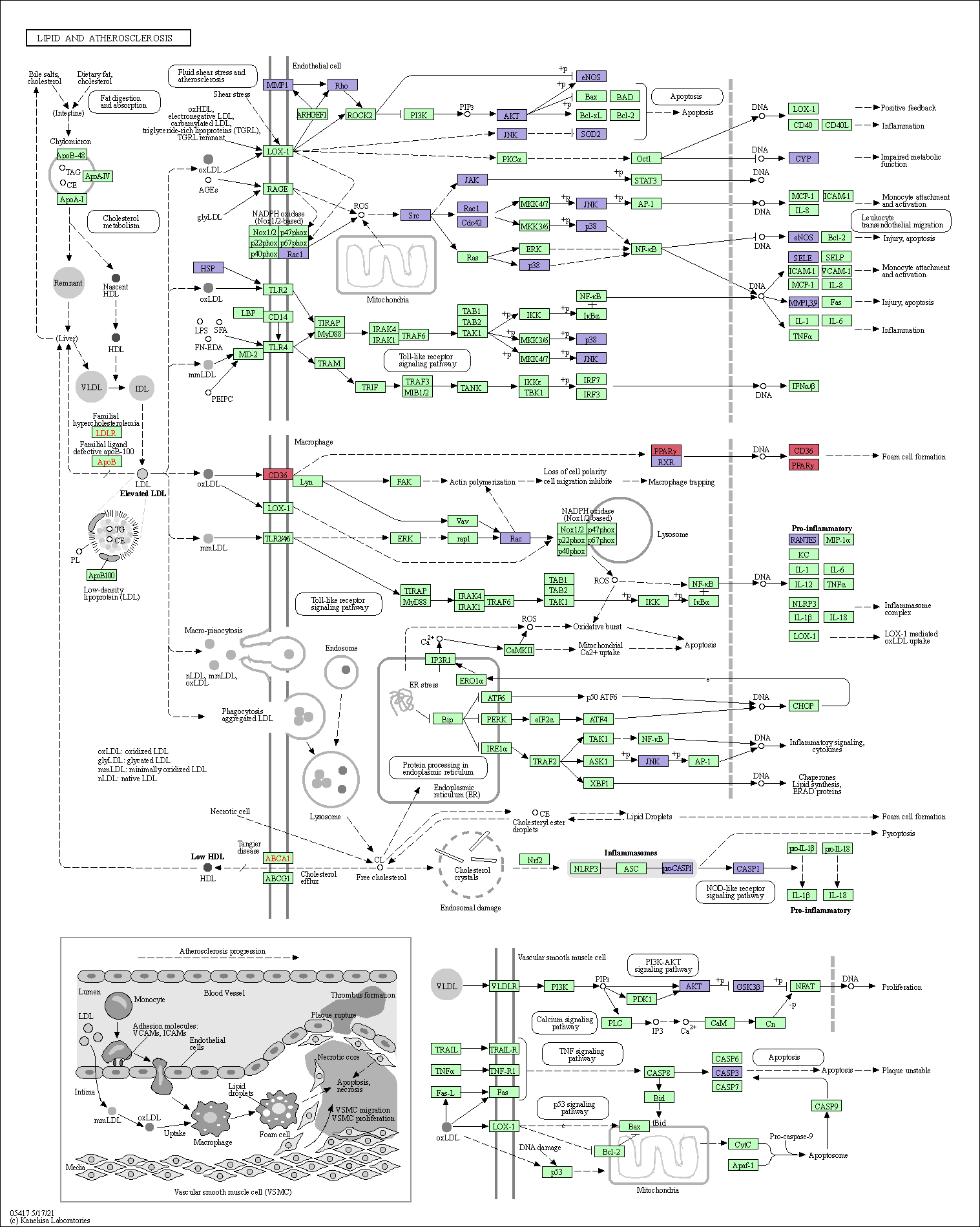

Supplement: Supplementary file 3 — Supplementary Material 3 [file 12944_2024_2049_MOESM3_ESM.tif]

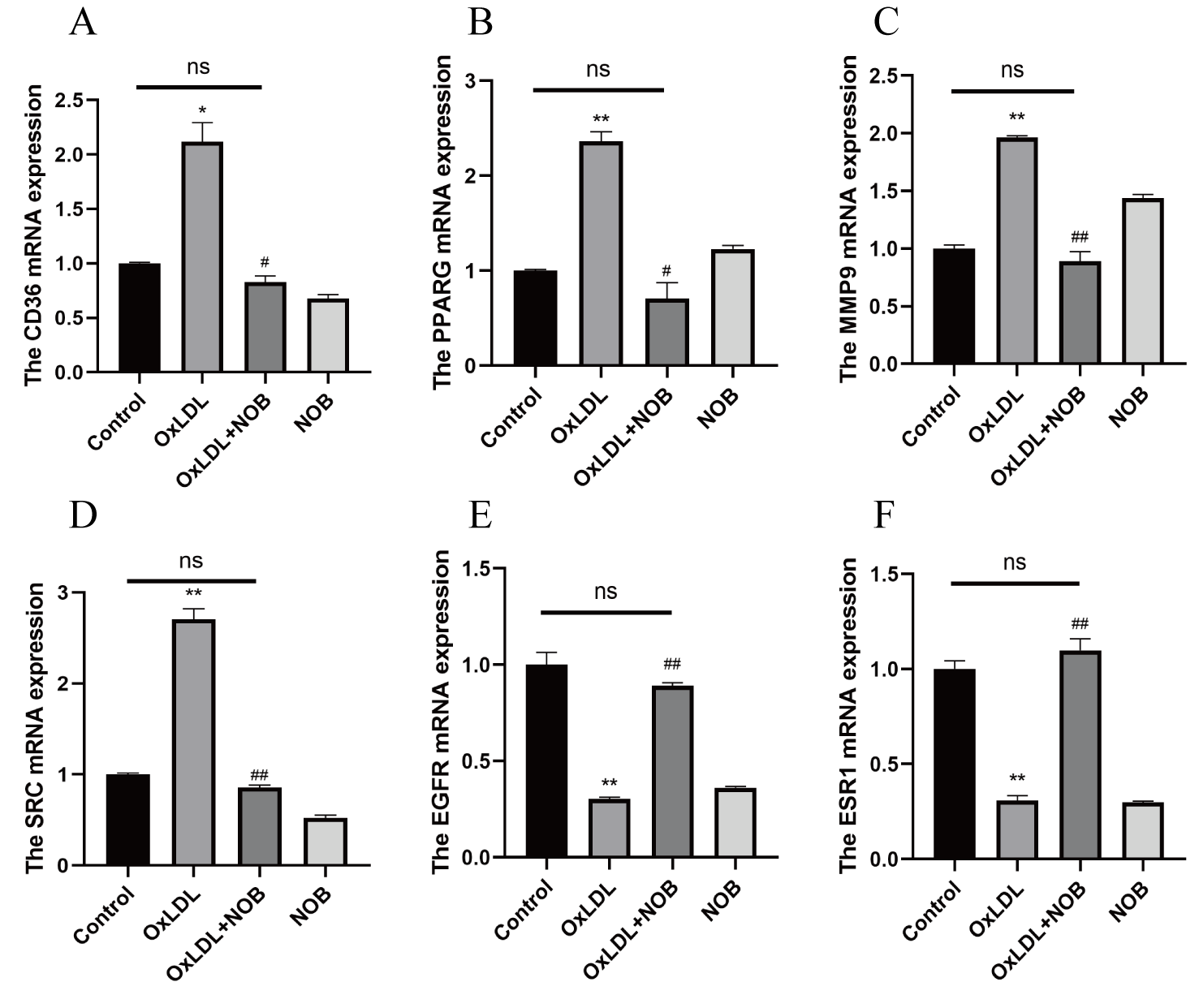

Supplement: Supplementary file 4 — Supplementary Material 4 [file 12944_2024_2049_MOESM4_ESM.tif]

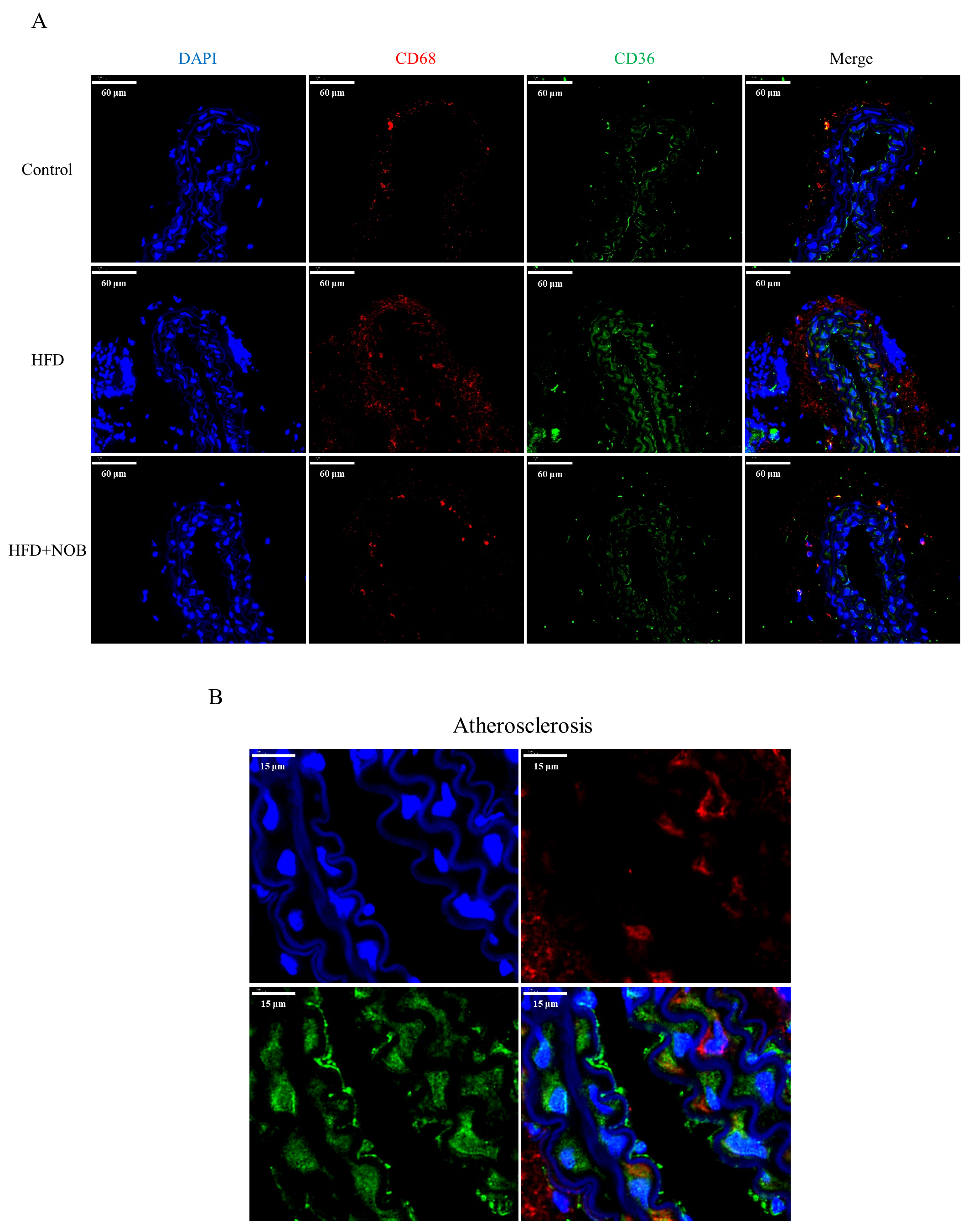

Supplement: Supplementary file 5 — Supplementary Material 5 [file 12944_2024_2049_MOESM5_ESM.tif]
